# 11-Ketotestosterone is the predominant active androgen in prostate cancer patients after castration

**DOI:** 10.1172/jci.insight.148507

**Published:** 2021-06-08

**Authors:** Gido Snaterse, Lisanne F. van Dessel, Job van Riet, Angela E. Taylor, Michelle van der Vlugt-Daane, Paul Hamberg, Ronald de Wit, Jenny A. Visser, Wiebke Arlt, Martijn P. Lolkema, Johannes Hofland

**Affiliations:** 1 Department of Internal Medicine, Section of Endocrinology, Erasmus MC, Rotterdam, Netherlands.; 2 Department of Medical Oncology, Erasmus MC Cancer Institute, Erasmus MC, Rotterdam, Netherlands.; 3 Institute of Metabolism and Systems Research, University of Birmingham, Birmingham, United Kingdom.; 4 Department of Internal Medicine, Franciscus Gasthuis & Vlietland, Rotterdam, Netherlands.

**Keywords:** Endocrinology, Oncology, Prostate cancer

## Abstract

**BACKGROUND:**

Continued androgen receptor (AR) signaling constitutes a key target for treatment in metastatic castration-resistant prostate cancer (CRPC). Studies have identified 11-ketotestosterone (11KT) as a potent AR agonist, but it is unknown if 11KT is present at physiologically relevant concentrations in patients with CRPC to drive AR activation. The goal of this study was to investigate the circulating steroid metabolome including all active androgens in patients with CRPC.

**METHODS:**

Patients with metastatic CRPC (*n* = 29) starting a new line of systemic therapy were included. Sequential plasma samples were obtained for measurement of circulating steroid concentrations by multisteroid profiling employing liquid chromatography–tandem mass spectrometry. Metastatic tumor biopsy samples were obtained at baseline and subjected to RNA sequencing.

**RESULTS:**

11KT was the most abundant circulating active androgen in 97% of patients with CRPC (median 0.39 nmol/L, range: 0.03–2.39 nmol/L), constituting 60% (IQR 43%–79%) of the total active androgen (TA) pool. Treatment with glucocorticoids reduced 11KT by 84% (49%–89%) and testosterone by 68% (38%–79%). Circulating TA concentrations at baseline were associated with a distinct intratumor gene expression signature comprising AR-regulated genes.

**CONCLUSION:**

The potent AR agonist 11KT is the predominant circulating active androgen in patients with CRPC and, therefore, one of the potential drivers of AR activation in CRPC. Assessment of androgen status should be extended to include 11KT, as current clinical approaches likely underestimate androgen abundance in patients with CRPC.

**TRIAL REGISTRATION:**

Netherlands Trial Register: NL5625 (NTR5732).

**FUNDING:**

Daniel den Hoed Foundation and Wellcome Trust (Investigator Award WT209492/Z/17/Z).

## Graphical abstract

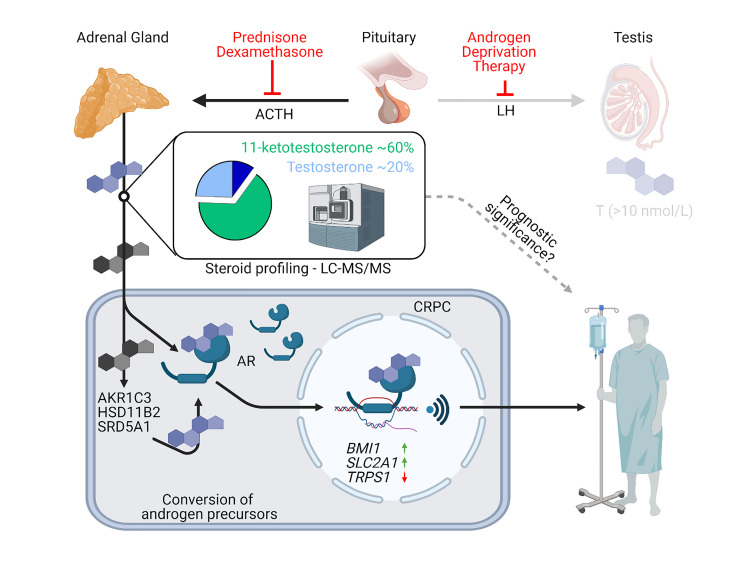

## Introduction

Targeting the androgen receptor (AR) pathway through androgen deprivation therapy (ADT) is the mainstay of treatment in metastatic prostate cancer (PC; ref. 1). Eventually, most tumors will evolve from hormone-sensitive to castration-resistant prostate cancer (CRPC) and show progression despite suppressed testosterone (T) levels. The continued importance of the AR pathway in tumor growth and progression has been underlined by the efficacy of novel drugs targeting the AR pathway (2–5). AR upregulation (6), increased intratumor conversion of adrenal androgen precursors (7–9), noncanonical dihydrotestosterone (DHT) biosynthesis (10), and downregulation of androgen-inactivating enzymes (11, 12) may all contribute to AR pathway reactivation.

In recent years, novel androgenic steroids have been identified with significant AR activation potential (13). The adrenal-derived steroid 11-ketotestosterone (11KT) is of particular interest because it is one of a few endogenous steroids capable of activating the AR at subnanomolar concentrations, similar to T and DHT (13–15). In healthy adult men, circulating T concentrations exceed those of 11KT (16, 17). During ADT, gonadal steroidogenesis is inhibited and T concentrations typically fall below 0.5 nmol/L (18), which is lower than 11KT concentrations in healthy men (16).

**Figure 1:**
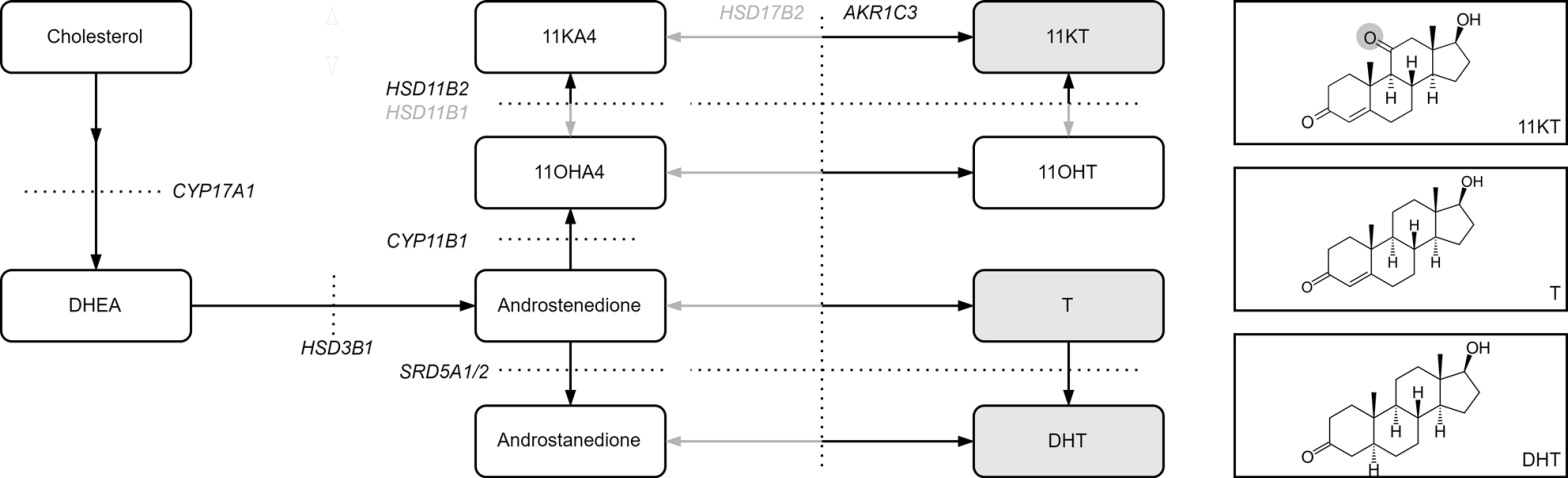
Androgen biosynthesis. Schematic overview of the conversion of adrenal precursor steroids to the potent androgens T, DHT, and 11KT. The molecular structures of the active androgens are shown, with the 11-keto group highlighted in gray. DHEA, dehydroepiandrosterone.

We hypothesized that due to the adrenal origin of its precursors, 11KT may persist after castration and may therefore exceed the residual concentrations of T and DHT in patients with CRPC. Similarly, the 11-oxygenated precursor steroids 11β-hydroxyandrostenedione (11OHA4), 11-ketoandrostenedione (11KA4), and 11β-hydroxytestosterone (11OHT), which can be converted into 11KT (Figure 1), may persist after castration and serve as substrate for intratumor androgen production. Androgen abundance after castration has been associated with improved response to AR pathway inhibition in patients with CRPC (19–21). Thus, persistence of a previously overlooked, potent androgen class would be of major clinical significance in patients with CRPC.

In this study, we carried out mass spectrometry–based multisteroid profiling in the plasma of 29 patients before, during, and after treatment for CRPC. We report the abundance of circulating active androgens in these patients. Additionally, we show the effects imposed on the steroid metabolome by treatment with exogenous glucocorticoids. Finally, we show that total androgen levels including 11KT are linked to differential gene expression in tumor biopsies.

## Results

### Patients.

**Figure 2:**
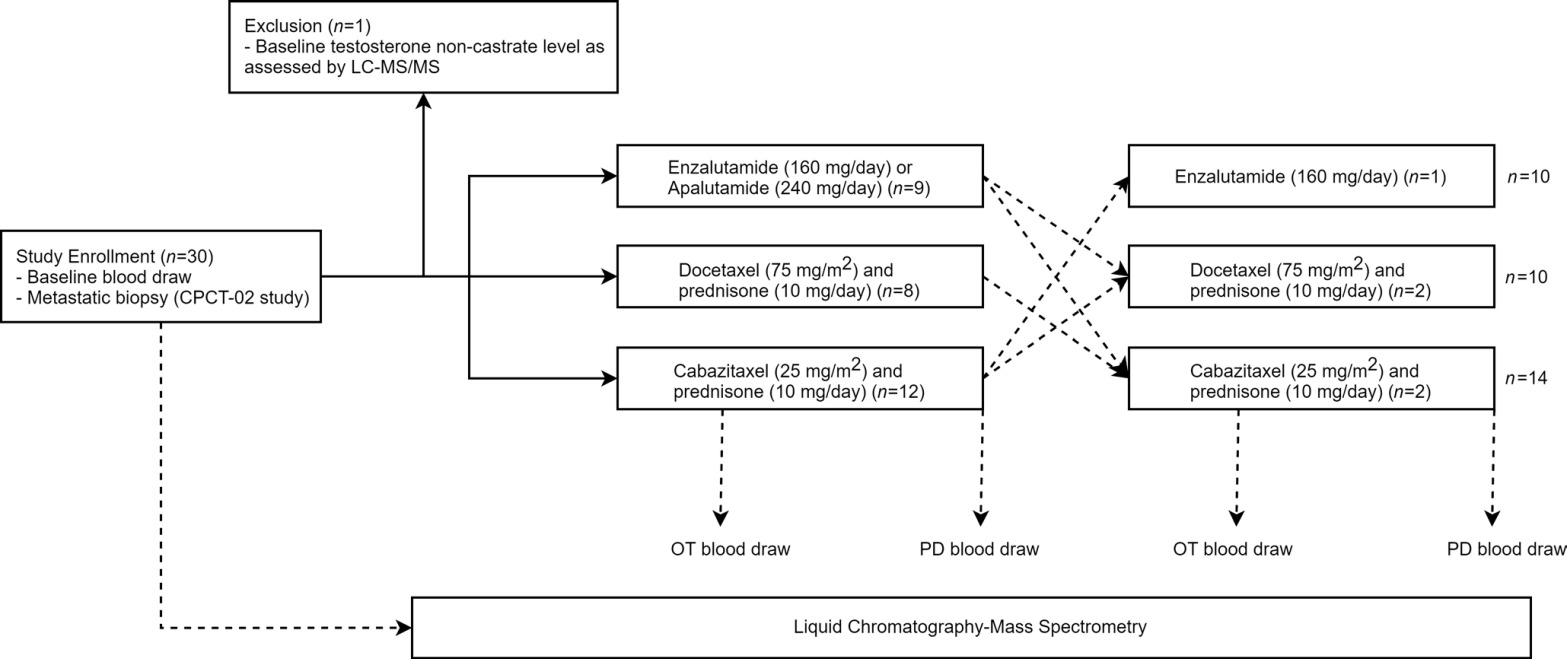
Patient and sample selection. Selection and exclusion of CIRCUS study samples for multisteroid profiling, glucocorticoid quantification, survival analysis, and tumor biopsy analysis. LC-MS/MS, liquid chromatography-tandem mass spectrometry.

Samples used in this study were obtained between May 2016 and July 2018 from 30 patients with metastatic CRPC who were scheduled to start a new line of treatment. One patient was excluded based on noncastrate T concentrations at baseline. In total, 29 patients who completed 34 treatments were included in our analysis (Figure 2); 5 patients completed 2 treatments during their enrollment. Patient characteristics, disease, and treatment history are shown in Table 1. For 5 patients with early progression (progression-free survival,PFS: 22–82 days) no separate OT sample was available. These subjects were included in the comparison between baseline and OT samples but not between OT and PD samples.

### Androgen abundance.

**Figure 3:**
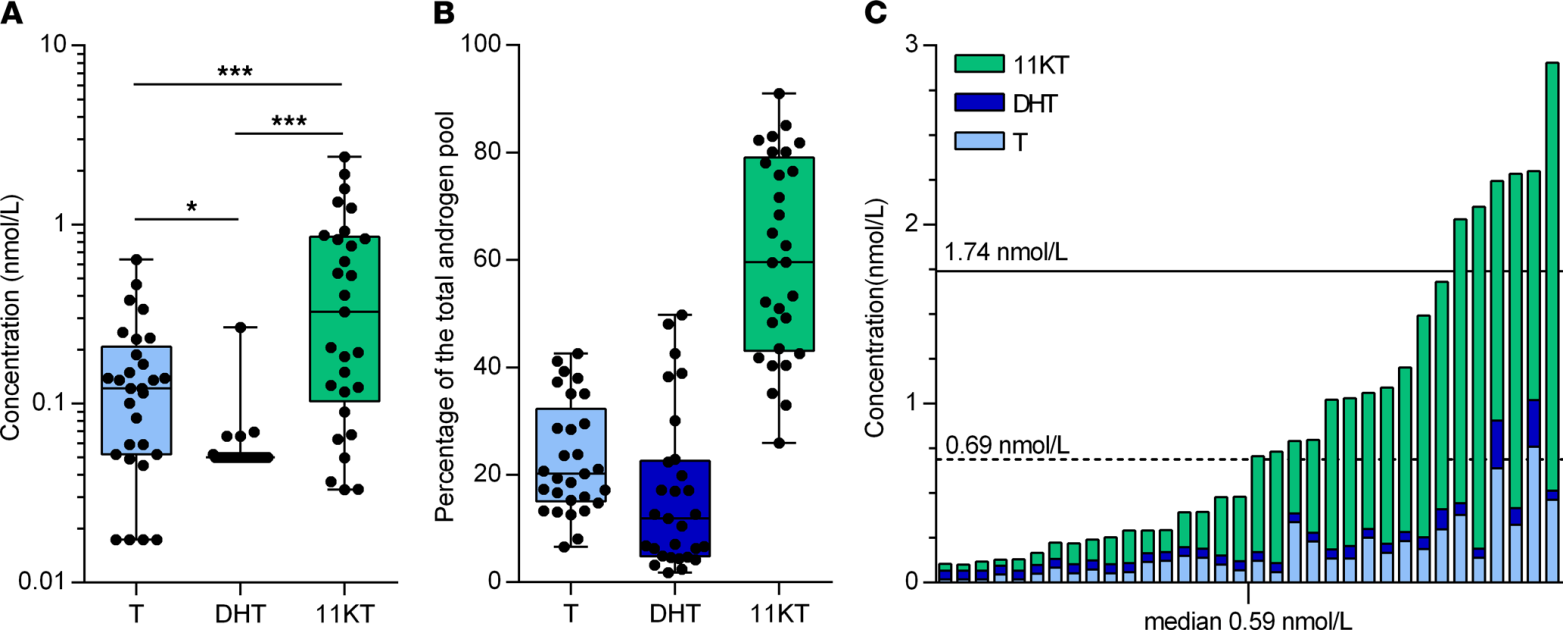
11-KT is the most abundant circulating active androgen in CRPC patients at baseline. (**A**) Active androgen concentrations of all patients with CRPC before the start of the first treatment after enrollment (*n* = 29). Box plot depicts the upper and lower quartiles, with the median shown as a solid line; whiskers indicate the range. Dots indicate individual data points. Statistical analysis was performed by 1-way ANOVA (*P* < 0.0001) with Tukey’s multiple-comparison test. *P < 0.05, ***P < 0.001. (**B**) The relative abundance of each androgen is shown as a percentage of the total androgen pool. Box plot depicts the upper and lower quartiles, with the median shown as a solid line; whiskers indicate the range. Dots indicate individual data points. (**C**) Active androgen concentrations are shown for all baseline samples (*n* = 34). Values below the analytical limit of quantification are shown if relevant calibrator and spiked quality control samples were accurate and reproducible with signal-to-noise ratio greater than 10:1. Samples with undetectable concentrations were set to 0.5 times the lowest accurate calibration sample for statistical purposes. Conventional clinical cutoff values for castrate testosterone levels (0.69 and 1.74 nmol/L, or 20 and 50 ng/dL, testosterone) are indicated on the *y* axis for reference. OT, on treatment; PD, progressive disease.

The circulating active androgen concentrations in patients with CRPC were determined by LC-MS/MS in samples obtained before the start of the first treatment after enrollment (*n* = 29). The median concentration of 11KT (0.33 nmol/L, range 0.03–2.39 nmol/L) was significantly higher than T (0.12 nmol/L; range 0.03–0.64 nmol/L, *P* < 0.001) in patients with CRPC at baseline (Figure 3A).

11KT constituted 60% (43–79%) of the total active androgen (TA) pool whereas T was 20% (15–32%; Figure 3B). Although T was below the castrate cutoff (1.74 nmol/L, 50 ng/mL) in all baseline samples (*n* = 34), the TA concentration (0.59 nmol/L, 0.23–1.27 nmol/L) exceeded 1.74 nmol/L in 6 patients (Figure 3C). DHT was below the lower limits of quantification (LOQ) in most patients (range: <LOQ–0.27 nmol/L). The correlation between most of the 11-oxygenated androgens was high (*R*^2^ = 0.71–0.87, respectively, Supplemental Figure 1; supplemental material available online with this article; https://doi.org/10.1172/jci.insight.148507DS1), with the exception of 11OHT (*R*^2^ = 0.39–0.55). Of the 11-oxygenated precursor steroids, 11OHA4 showed the closest correlation with testosterone (*R*^2^ = 0.73), 11KT (*R*^2^ = 0.87), and TA (*R*^2^ = 0.86).

### Effects of treatment.

Subjects included in this study started treatment with AR antagonists (*n* = 10), docetaxel with prednisone (*n* = 10), or cabazitaxel with prednisone (*n* = 14). Steroid hormone concentrations at baseline stratified for the different treatments are shown in Supplemental Figure 2, A–D. Significant suppression of adrenal-derived steroids was observed after 12 weeks of cabazitaxel with prednisone treatment. In the docetaxel group, a similar suppression was observed in a subset of patients, but overall, this suppressive effect did not reach significance. In the AR antagonist group, increased steroid concentrations were observed after 12 weeks of treatment.

Low baseline cortisol concentrations were detected in a subset of patients, suggestive of hypothalamus-pituitary-adrenal (HPA) axis suppression by exogenous glucocorticoids. Post hoc exogenous glucocorticoid quantification by LC-MS/MS was performed to detect prednisone, prednisolone, and dexamethasone in all samples (Supplemental Figure 3, A–C). Samples were classified as exogenous glucocorticoid positive if prednisolone (≥20.7 ng/mL) and/or dexamethasone (≥16.1 ng/mL) were detected. Cortisol was suppressed (<140 nmol/L) in all exogenous glucocorticoid–positive baseline samples (Supplemental Figure 3D).

**Figure 4:**
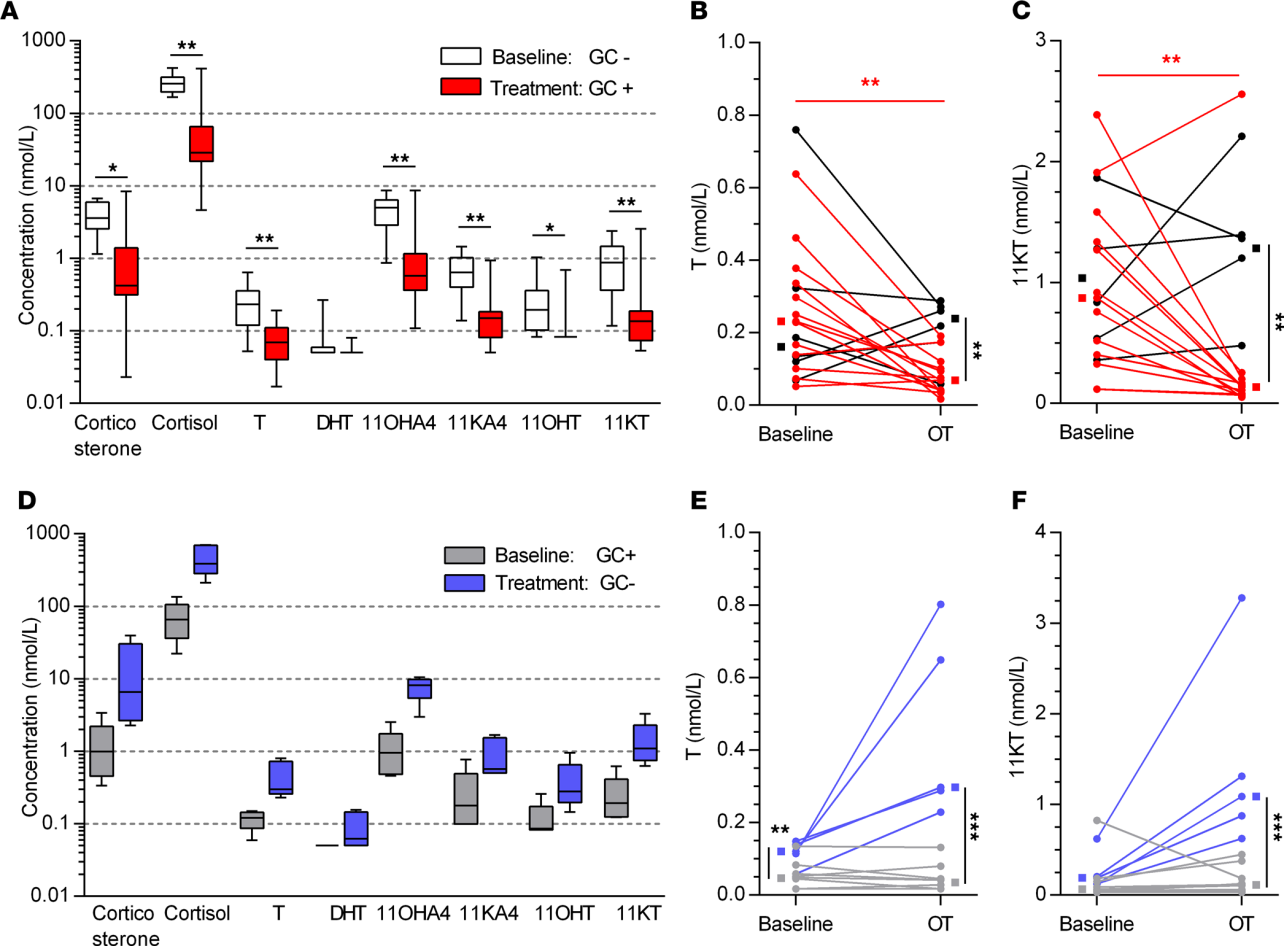
Effects of exogenous glucocorticoid treatment on circulating steroid concentrations. (**A**) Differences between steroid concentrations at baseline (white boxes) and OT (red boxes) were assessed in patients who were exogenous glucocorticoid untreated at baseline and who started treatment with glucocorticoids (*n* = 13) by Wilcoxon’s signed-rank test. The individual data points are shown for (**B**) T and (**C**) 11KT at baseline and OT in patients who started therapy with glucocorticoids (red lines, *n =* 13) or without glucocorticoids (black lines, *n* = 6). (**D**) Differences between concentrations at baseline (gray boxes) and OT (blue boxes) were assessed in patients who were glucocorticoid treated at baseline and discontinued glucocorticoid treatment (*n* = 5). The individual data points are shown for (**E**) T and (**F**) 11KT at baseline and OT in patients who continued treatment with glucocorticoids (gray lines, *n* = 10) or were withdrawn from glucocorticoids (blue lines, *n* = 5). Box plot depicts the upper and lower quartiles, with the median shown as a solid line; whiskers indicate the range. Effects of treatment were assessed by Wilcoxon’s rank-sum test, while group differences were assessed by Mann-Whitney *U* test. Lines connect individual patients and group medians (squares) are shown beside the individual data points. *P < 0.05, **P < 0.01, ***P < 0.001.

A significant reduction in circulating glucocorticoids as well as T and 11KT concentrations was observed in patients treated with exogenous glucocorticoids (Figure 4A). Circulating T (Figure 4B) and 11KT (Figure 4C) concentrations were lowered by 68% (IQR: 38–79%) and 84% (49–89%), respectively, in patients starting exogenous glucocorticoids. Decreases of similar magnitude were observed for 11OHA4, 11OHT, and 11KA4 (medians 66%–92%; Figure 4A). In a subset of exogenous glucocorticoid–treated patients, glucocorticoid treatment was withdrawn. The group size was insufficient to detect a statistical difference between baseline and treatment (*n* = 5, Figure 4D). However, compared with patients who continued exogenous glucocorticoid treatment (*n* = 10), withdrawn patients had 8-fold higher T (0.30 nmol/L [IQR 0.26–0.73 nmol/L] vs. 0.04 nmol/L [0.02–0.05 nmol/L]) and 10-fold higher 11KT (1.09 nmol/L [IQR 0.75–2.30 nmol/L] vs. 0.11 nmol/L [0.04–0.23 nmol/L]) (Figure 4, E and F). An overview of steroid concentrations at baseline can be found in Supplemental Table 1. Additionally, exogenous glucocorticoid treatment was withdrawn in 6 patients before progression. Again, higher median circulating concentrations of T (0.20 nmol/L [0.09–0.38 nmol/L] vs. 0.05 nmol/L [0.02–0.08 nmol/L], *P* < 0.01) and higher median 11KT (0.90 nmol/L [0.52–1.46 nmol/L] vs. 0.10 nmol/L [0.06–0.29 nmol/L], *P* = 0.001) were observed after withdrawal, compared with patients who continued glucocorticoid treatment (*n* = 14, Supplemental Figure 4).

### Clinical outcomes.

**Figure 5:**
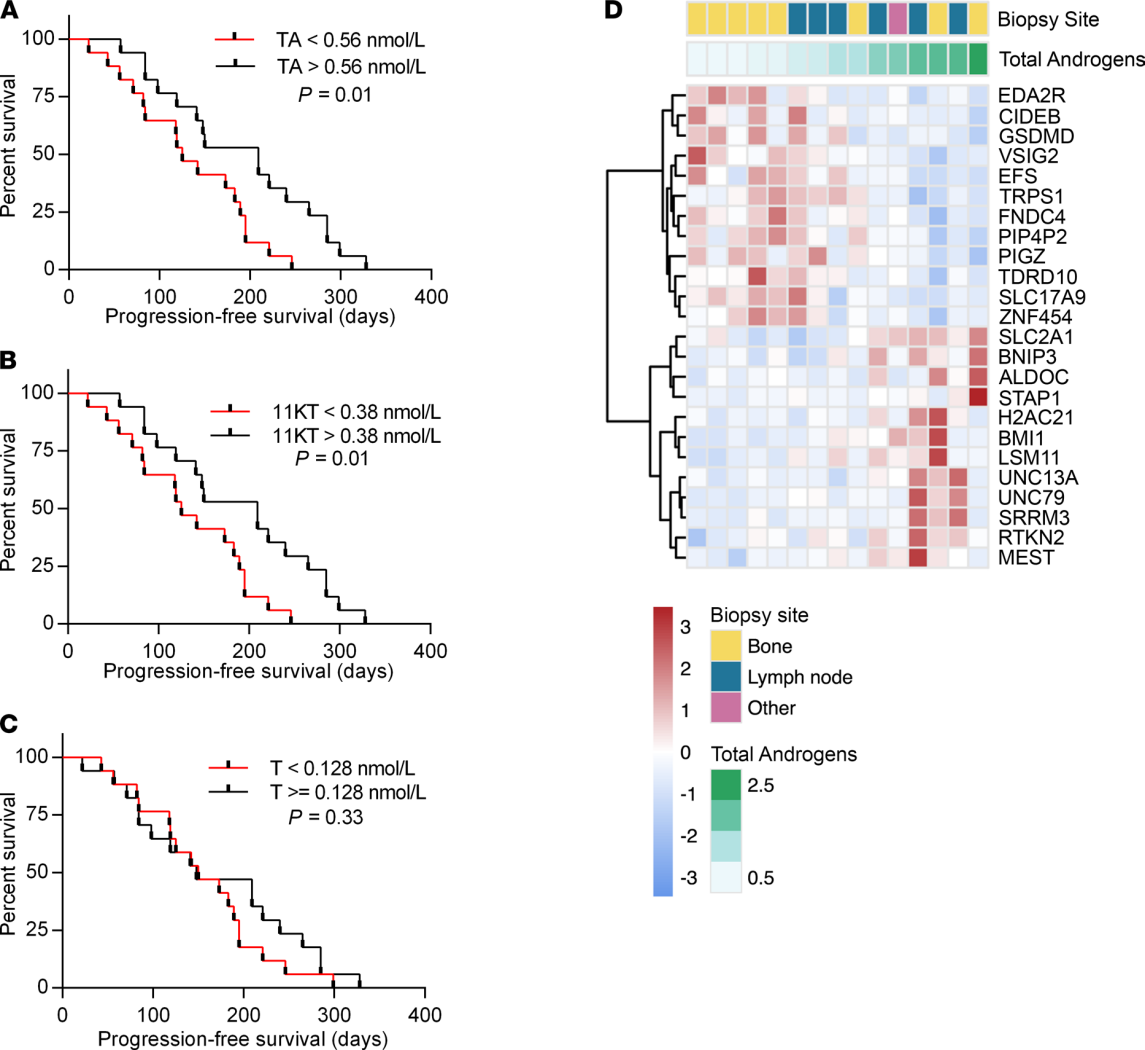
Effects of TA concentration on PFS and intratumor gene expression. PFS curves are shown for patients stratified into 2 groups with concentrations above or below median (**A**) TA (defined as the sum of T, DHT, and 11KT), (**B**) 11KT, or (**C**) T. Log-rank test for survival was used to determine difference between the low- and high-TA groups. (**D**) Heatmap of differentially expressed genes (*n* = 24) across TA concentration in the tumor samples. Differential gene expression was determined using TA concentration as a continuous variable. Heatmap displays mean-centered and normalized (variance-stabilizing transformation) read counts. Unsupervised hierarchical clustering (Euclidean distance and Ward.D2 method) was performed on genes and samples. Upper tracks display biopsy site and TA concentrations.

A post hoc survival analysis was performed on this limited patient group. High TA concentrations (above the median) associated with a longer PFS in patients (209 vs. 133.5 days, *P* < 0.05, Figure 5A). Stratification based on 11KT alone similarly showed this association (Figure 5B), whereas T alone did not (Figure 5C). None of the 11-oxygenated androgen precursors was independently associated with survival (Supplemental Figure 5). Overall survival was not affected by TA pool quantities (14.7 months vs. 12.3 months, *P* > 0.05).

### RNA-sequencing analysis.

Gene expression profile analysis of the complete CPCT-02 CRPC cohort (*n* = 180) revealed significant biopsy site–related bias, which we attempted to limit through exclusion of 5232 biopsy site–specific genes (Supplemental Figure 6). Next, biopsy material obtained from 15 of the patients included in this study was assessed by RNA sequencing, excluding genes that were biopsy site related (Supplemental Figure 7). Using TA concentration as a continuous variable, we observed androgen-mediated differential expression of 24 genes (Figure 5D), including several known androgen-regulated genes. Of those, 12 were upregulated in the high-TA environment, including the known AR target genes *BMI1* (22) and *SLC2A1* (23), and the other 12 in the low-TA environment, including the androgen-repressed gene *TRPS1* (24). Furthermore, a trend toward increased *AR* expression in the low-TA environment (5.4-fold higher) was observed, although this did not reach statistical significance.

Several enzymes related to androgen biosynthesis and (re-)activation were highly expressed in all tumor biopsy samples, including *HSD17B10*, *STS*, *SRD5A1*, *AKR1C3*, and *HSD11B2* (Supplemental Figure 8). This suggests that the enzymes required for activation of both androgen and 11-oxygenated androgen precursors are expressed in CRPC tumors.

## Discussion

Using mass spectrometry–based plasma multisteroid profiling, we show that the potent AR agonist 11KT is the predominant circulating active androgen in patients with CRPC and therefore needs to be considered when assessing the hormonal status of these patients. 11KT constituted a median 60% of the active androgen pool, signifying that androgen abundance in patients with CRPC is currently systematically underestimated. Estimation of the TA abundance after castration could be assessed more accurately by including 11KT. Additionally, this study shows the suppressive effects of exogenous glucocorticoids on circulating androgen concentrations, highlighting the potential therapeutic role of glucocorticoids in CRPC, which crucially includes suppression of adrenal-derived 11-oxygenated androgens.

AR pathway reactivation after ADT is an important process that leads to tumor progression. In most patients, 11KT was more abundant than T and DHT combined, and our data suggest that 11KT may be an important contributor to AR reactivation in CRPC. In addition to direct activation of the AR by active androgens, intratumor conversion of precursor steroids into active androgens may further contribute to AR reactivation. Storbeck et al. (25) previously showed that the PC cell line LNCaP converts 11OHA4 and 11OHT into 11KT, requiring the enzymes HSD11B2 and AKR1C3. We confirm substantial expression of *HSD11B2* and *AKR1C3* in nearly all CRPC tumor samples (93% and 100%, respectively). Expression of enzymes that inactivate (11-oxygenated) androgens to their upstream precursors, such as *HSD17B2* and *HSD11B1*, was lower. A higher ARK1C3/HSD17B2 ratio favors production of 11KT especially, as AKR1C3 has a significantly higher substrate preference for 11KA4 than androstenedione (26). The intratumor androgen levels could not be studied in this study, however, and this is necessary to fully elucidate the role of the 11-oxygenated androgen precursors.

We found that the 11-oxygenated androgen precursors 11OHA4 and 11KA4 persisted after castration and correlated with 11KT. The concentrations detected in our patients without exogenous glucocorticoid treatment are comparable to previously reported concentrations of 11OHA4 and 11KA4 in elderly men (17). In line with previous reports, we found that 11OHA4 was the most abundant 11-oxygenated androgen in the circulation, with a median concentration of 2.7 nmol/L (0.48–5.1 nmol/L) in all patients with CRPC and 4.96 nmol/L (3.05–6.13 nmol/L) in patients who did not receive exogenous glucocorticoid treatment. 11OHT was the least abundant of the 11-oxygenated androgens studied. Due to the low concentrations and relatively lower affinity for the AR (25), it was not included in our calculation of the TA pool. Androstenedione could not be quantified in our study due to a technical limitation, but the concentration in CRPC patients has been determined in earlier studies (18).

Glucocorticoid treatment significantly decreased circulating T (*P* < 0.01) and 11-oxygenated androgens (*P* < 0.05 and *P* < 0.01, respectively) through suppression of the HPA axis. Potent suppression of 11-oxygenated androgens has previously been observed in patients treated with abiraterone acetate and prednisone (27). Abiraterone acetate suppresses the production of adrenal androgens through direct inhibition of CYP17A1, rather than negative feedback. Prednisone is administered to these patients to prevent increased production of adrenocorticotropic hormone, which may otherwise lead to mineralocorticoid excess. The potent suppression of adrenal androgens, and 11KT in particular, may partially explain the clinical benefits of glucocorticoid treatment independent of abiraterone and should be considered when designing trials with glucocorticoid treatment in the control arm (28, 29). Withdrawal of glucocorticoid treatment may inadvertently lead to an increase in circulating androgens, although the effect on CRPC tumor growth is still unknown.

Gene expression analysis of the tumor biopsy samples in a subset of patients identified 24 genes differentially expressed between patients across the TA spectrum, including 2 androgen-stimulated genes and 1 androgen-repressed gene (22–24). *TRPS1* and *SLC2A1* have previously been implicated in PC and AR action (30, 31), and decreased *EFS* expression has been associated with more advanced PC and tumor recurrence (32, 33). Other genes differentially expressed in our cohort (*EDA2R*, *SLC17A9*, *TDRD10*, *ALDOC*, *SRRM3*, *MEST*, and *RTKN2*) have been implicated in other malignancies but not in PC specifically (34–38).

PFS was longer in our patients with high TA, in line with earlier findings that higher T concentrations at the CRPC stage are associated with a more favorable response to AR pathway inhibition (19–21). Interestingly, in our study this association was attributed to 11KT, as T alone was not associated with PFS. In another study, improved outcome in docetaxel with prednisone-treated CRPC patients was associated with androstenedione (39). Although androstenedione could not be quantified in this study, it has been previously shown that androstenedione levels correlate with the levels of 11OHA4 and 11KT (40). Together, these findings indicate that the adrenal androgens and precursor steroids should be further investigated as potential prognostic markers. We hypothesize that while adrenal androgens persist, cancer cells that rely on intratumor conversion of precursors and ligand-dependent AR activation may have a competitive advantage, resulting in a selection for cells that inadvertently remain responsive to conventional treatments that target the AR signaling pathway. However, AR pathway inhibition may result in the development of androgen-independent resistance mechanisms, such as expression of AR variants (41), expression of the glucocorticoid receptor (42), or development of a neuroendocrine phenotype (43), which is associated with poor prognosis (44, 45). CRPC cells in lower androgen milieu may be under increased selective pressure for adverse disease characteristics, contributing to lower PFS. It is, however, important to consider that our study was not designed to detect differences in survival, and the limited sample size and patient heterogeneity do not permit a definitive conclusion in this regard. Most patients in the CIRCUS study had previously received treatment for CRPC. No association between the number of treatment lines and PFS was found, nor was the number of treatment lines different between the low- and high-TA groups. Further investigation into the actions and consequences of circulating 11KT is warranted, especially as a potential biomarker to select patients more likely to respond to AR-targeting therapies.

This study demonstrates that 11KT is the predominant circulating active androgen in patients with CRPC. Paired with evidence from previously published findings, our results position 11KT as one of the potential drivers of AR activation in CRPC. Both T and 11KT can be suppressed by glucocorticoid treatment, providing a possible explanation why glucocorticoids are beneficial in patients with CRPC. Future studies should consider the TA pool as a potential biomarker in patients who have undergone ADT.

## Methods

### Patients and samples.

From April 2016 onward, patients with metastatic CRPC who continued ADT and intended to start a new line of systemic therapy were included in the CIRCUS study (Netherlands Trial Registry ID: NL5625). Metastatic disease and progression were defined according to the Prostate Cancer Clinical Trials Working Group and/or Response Evaluation Criteria in Solid Tumors 1.1 criteria (46, 47). All patients who started treatment with AR antagonists (enzalutamide or apalutamide), docetaxel with prednisone, or cabazitaxel with prednisone were included in our analysis if blood samples were available both at baseline and upon progression (Figure 2). Concurrent participation in the CPCT-02 study (NCT01855477) was required to obtain a tumor biopsy before the start of therapy. Blood was collected in 3 CellSave preservative tubes (Menarini Sillicon Biosystems Inc) every 3 to 4 weeks, including at baseline, OT, and at PD. Plasma was isolated and stored as previously described (48, 49).

### Measurement of circulating steroids.

Calibration series (0.01–100 ng/mL) were prepared in duplicate in phosphate-buffered saline with 0.1% bovine serum albumin and in charcoal-stripped human serum (Goldenwest Diagnostics). Extraction and quantification of steroids were performed as previously described (40, 49–52). Briefly, steroids were obtained from 400 μL plasma by liquid-liquid extraction. Multisteroid profiling was performed by LC-MS/MS (Xevo TQ-XS) after separation on an ACQUITY UPLC (Waters) with UPLC HSS T3 column (21 mm × 50 mm, 1.8 μm, Waters). A representative chromatogram is shown in Supplemental Figure 9, and an overview of included steroids, their mass transitions, internal standards, and LOQ can be found in Supplemental Table 2. The Supplemental Methods section contains additional information regarding extraction, accuracy, precision, and values below LOQ. Masslynx (v4.1, Waters) was used to process LC-MS/MS data. The TA pool was defined as the sum of T, DHT, and 11KT concentrations because these steroids exert strong agonistic activity at the AR (13).

### RNA sequencing.

As part of the CPCT-02 study (NCT01855477), metastatic CRPC biopsy samples of patients included in this analysis were obtained at baseline. RNA sequencing was performed according to the manufacturer protocols using a minimum of 100 ng total RNA input. Total RNA was extracted using the QIAGEN QIAsymphony kit (FRITSCH GmbH). Paired-end sequencing of (m)RNA was performed on the Illumina NextSeq 550 platform (2 × 75 bp; Illumina) and NovaSeq 6000 platform (2 × 150 bp; Illumina). Downstream data processing and analysis are detailed in the Supplemental Methods. Briefly, CRPC tumor biopsies from the entire CPCT-02 study (*n* = 180) were used to identify biopsy site–specific genes. Subsequently, an untargeted approach was used to analyze gene expression across TA concentrations in biopsy samples of patients included in this study only (*n* = 15), excluding biopsy site–related genes. The expression of steroid hormone receptors and genes involved in steroid metabolism was assessed using a targeted approach (53).

### Data availability.

The LC-MS/MS data sets generated during and/or analyzed during the current study are available from the corresponding author. The presented whole-transcriptome sequencing data (.fastq) and corresponding attributes have been requested from Hartwig Medical Foundation and were provided under data request number DR-071 (February 2020). Both whole-transcriptome sequencing and clinical data are freely available for academic use from the Hartwig Medical Foundation through standardized procedures, and request forms can be found at https://www.hartwigmedicalfoundation.nl (54).

### Statistics.

Statistical analyses were performed with GraphPad Prism (version 6.01), SPSS (version 26, IBM Corp), and R (version 3.6.1). Logarithmic transformation was applied if obtained steroid concentrations did not pass D’Agostino and Pearson’s test for normality. We performed 1-way ANOVA with post hoc Dunnett’s test to compare circulating androgen concentrations. Wilcoxon’s signed-rank test was performed to assess the effects of treatment. Mann-Whitney *U* tests were used to compare the difference between exogenous glucocorticoid–treated and –untreated patients after 12 weeks of treatment. Linear models were used to assess how the individual active androgens were associated with TA. The associations between PFS and androgens were investigated at baseline and during treatment by the Kaplan-Meier method and differences compared by log-rank test. Correlation (Pearson) between androgens was determined after logarithmic transformation of baseline values. Group steroid concentrations and changes were reported as median with IQR, unless stated otherwise. A *P* value less than 0.05 was considered significant.

### Study approval.

The CIRCUS study (Netherlands Trial Registry ID: NL5625) was approved by the medical ethics board of the Erasmus MC (MEC-2016-081). All patients provided written informed consent before any study procedure; this involved blood collection at baseline, OT, and at PD and collection of clinical data.

## Author contributions

RDW, JAV, WA, MPL, and JH were responsible for study concept and design. LFVD, PH, RDW, and MPL recruited patients. All authors acquired, analyzed, or interpreted data. GS, LFVD, and JVR drafted the manuscript. All authors critically revised the manuscript. GS, LFVD, and JVR were responsible for statistics.JH and WA obtained funding. AET and MVDVD provided administrative, technical, or material support. JAV, MPL, and JH supervised the study. All authors reviewed and approved the final version of the manuscript. These authors contributed equally: GS and LFVD. GS was selected as the first of the 2 authors because he was responsible for the LC-MS/MS experiments. JH had full access to all the data in the study and takes responsibility for the integrity of the data and the accuracy of the data analysis.

## Supplementary Material

Supplemental data

## Figures and Tables

**Table 1:**
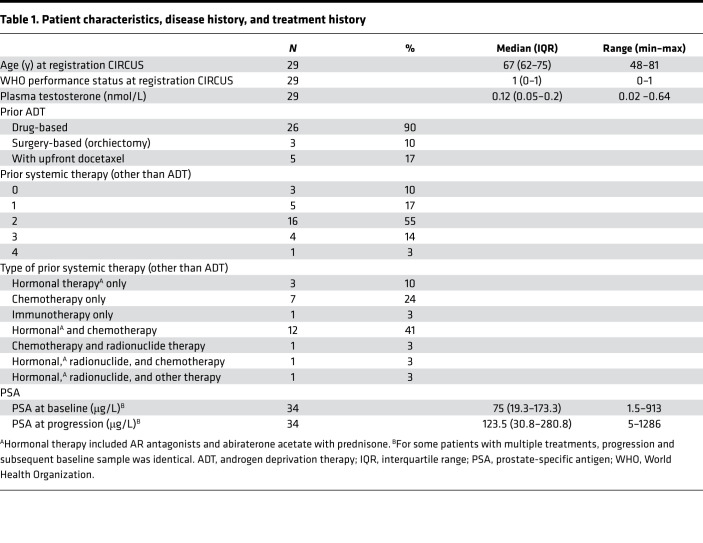
Patient characteristics, disease history, and treatment history

## Version 1 05/11/2021

In-Press Preview

## Version 2 06/08/2021

Electronic publication
