# Supplementary material for: 11-Ketotestosterone is the predominant active androgen in prostate cancer patients after castration
Source: JCI Insight. 2021 Jun 8;6(11):e148507. doi: 10.1172/jci.insight.148507 (PMC8262344; doi:10.1172/jci.insight.148507)
Supplement: Supplemental data [file jciinsight-6-148507-s234.pdf]

11-Ketotestosterone: the resilience of a potent androgen in prostate cancer patients after castration

- Online Only supplemental material -

Correspondence: Johannes Hofland

E-mail: j.hofland@erasmusmc.nl

Content:

- Supplementary Method
- Supplemental Table T1: **Baseline steroid concentrations of patients with or without exogenous glucocorticoids.**
- Supplemental Table T2: **Overview of steroids and their mass transitions, retention times and analytical limits of quantification.**
- Supplemental Figure S1: **Association between circulating androgens and 11-oxygenated androgens in CRPC patients at baseline**
- Supplemental Figure S2: **Baseline circulating steroid concentrations and effects of 12-week treatment**.
- Supplemental Figure S3: **Quantification of prednisone, prednisolone and dexamethasone identifies glucocorticoid suppressed patient samples.**
- Supplemental Figure S4: **Circulating androgens are higher in patients withdrawn before progression**.
- Supplemental Figure S5: **11-oxygenated androgen precursors are not associated with progression-free survival.**
- Supplemental Figure S6: **Overview of the CPCT-02 (m)RNA-sequenced metastatic prostate cancers (*n*= 180) including selection of biopsy site-specific expression**.
- Supplemental Figure S7: **Overview of the CPCT-02 (m)RNA-sequenced metastatic prostate cancers (n=15) present in the *CIRCUS* study.**
- Supplemental Figure S8: **Intratumoral expression of genes involved in steroid metabolism and steroid hormone receptors in metastatic castration-resistant prostate cancer.**
- Supplemental Figure S9: **Chromatographic separation of endogenous steroids**.

Supplemental Method

Extraction and measurement of endogenous steroids

Calibration series (0.01–100 ng/mL) were prepared in (1) phosphate-buffered saline + bovine serum albumin (0.1%) and in (2) charcoal-stripped human serum (Goldenwest Diagnostics, Temecula, CA, USA). Steroids were extracted from 400 µL plasma by liquid-liquid extraction using methyl-tert-butyl ether (MTBE, Sigma Aldrich, Zwijndrecht, the Netherlands) and evaporated under a nitrogen manifold at 50°C. Samples were reconstituted in 125 µL LC-MS grade 50% methanol (CHROMASOLV, Sigma Aldrich). Multi-steroid profiling was performed by tandem mass spectrometry (Xevo TQ-XS, Waters, Milford, MA, USA) after separation on an ACQUITY UPLC (Waters) with UPLC high-strength silica T3 column (21 mm x 50 mm, 1.8 µm, Waters) as previously described(1-5). Spiked analytical controls showed accuracy of ≥90% at very low (0.03 ng/mL), low (0.3 ng/mL), medium (3 ng/mL) and high (30 ng/mL) concentrations for testosterone, 11KT, 11KA4 and cortisone. For 11OHA4, controls showed accuracy ≥80% at very low (0.03 ng/mL) and ≥90% at higher concentrations. Cortisol, corticosterone, DHT and 11OHT showed accuracy of ≥90% at low to high concentrations. Inter-assay precision was calculated based on 4-6 biological replicates ran on 3 days, with RSD <10% for testosterone, DHT, 11KT, cortisol, cortisone and corticosterone, <20% for 11OHT, and 22% -for 11OHA4 and 32% for 11KA4. Calibration of androstenedione was not successful due to a matrix contaminant and was excluded from further analysis.

Data has been included where steroid concentrations were below the analytical LOQ, but where calibration and spiked QC samples were still accurate with signal to noise >10:1. Samples with undetectable concentrations were set to 0.5 times the lowest accurate calibration point for statistical purposes. The total active androgen (TA) pool was defined as the sum of testosterone, DHT and 11KT as these steroids have been confirmed to directly activate the AR(6).

Quantification of exogenous glucocorticoids

Exogenous glucocorticoids were measured in the original sample plates by LC-MS/MS using a protocol optimized for separation of the exogenous and endogenous glucocorticoids(7, 8). To quantify the steroids a calibration series (1 – 100 ng/mL) containing prednisone, prednisolone and dexamethasone was prepared. Cutoff values for prednisolone (20.7 ng/mL) and dexamethasone (16.1 ng/mL) were determined based on suppression of cortisol (<140 nmol/L) and were used to distinguish exogenous glucocorticoid-treated and untreated samples.

RNA sequencing

Sequencing libraries were prepared using the KAPA RNA HyperPrep Kit with RiboErase (HMR) KR1351 (Roche, Indianapolis, IN) according to the manufacturer protocols. Paired-end sequencing of (m)RNA was performed on the Illumina NextSeq 550 platform (2x75bp; Illumina, San Diego, California, USA) and Illumina NovaSeq 6000 platform (2x150bp; Illumina, San Diego, California, USA). Raw sequencing reads were trimmed (paired-end) with fastp(9) (v0.20.0) using default settings to perform adapter, low-quality and low-complexity trimming using the following command:

```
fastp --detect_adapter_for_pe -L --html --thread 5 --in1 <fq.R1> --in2 <fq.R2> --out1
<fq.R1.out> --out2 <fq.R1.out>
```

Trimmed paired-end reads were subsequently aligned against the human genome reference build 37 (GRCh37) with STAR(10) (v2.7.3a) on GENCODE annotations(11) (v33). Samples sequenced on multiple sequencing lanes were aligned simultaneously with respective read-group information using the following command:

```
STAR --genomeDir <GRCh37> --readFilesIn <fq.R1> <fq.R2> --readFilesCommand zcat --
outFileNamePrefix <prefix> --outSAMtype BAM SortedByCoordinate --outSAMunmapped Within --
```

```
twopassMode Basic --twopass1readsN -1 --runThreadN 10 --limitBAMsortRAM 10000000000 --
quantMode TranscriptomeSAM --outSAMattrRGline <readgroup>
```

Marking of duplicate reads, sorting, indexing and retrieving flagstat information were performed using Sambamba(12) (v0.7.1; **Supplemental Figure S6A**). Overlapping primary-aligned reads per exon were summarized per gene by Subread featurecounts(12) (v1.6.3) using reversely-stranded modus on GENCODE annotations (v33):

```
featureCounts -T 50 -t exon -g gene_id --primary -p -s 2 -a <gencodev33.gtf> -o <output> <BAM
files>
```

Read counts from protein coding genes present in the GENCODE annotations (v33; n = 20084) were inputted into DESeq2(13) (v1.24.0) for all 180 RNA-sequenced samples and normalized for library-size using default settings.

To identify possible batch-effects in expression due to biopsy localization (**Supplemental Figure S6B**) or sequencing platform (Illumina NextSeq / Illumina NovaSeq), we first performed t-SNE(14) analysis using Rtsne (v0.15; with Θ set to 0.5, a perplexity of 30, 1000 iterations and two dimensions) on normalized reads counts (variance stabilizing transformation (VST)) to reveal signs of possible batch-effects (**Supplemental Figure S6D**). This analysis revealed several clusters attributed to biopsy location and only limited correlation to sequencing technique. To reduce the batch-effect seen by biopsy localization, we performed differential analysis using DESeq2 (v1.24.0; Wald method) with additional LFC shrinkage (ashr(15); v2.2.47) per major biopsy site and comparing against all others (**Supplemental Figure S6B**); bone (n = 89 vs. 91), lymph node (n = 42 vs. 138) and liver (n = 31 vs. 149). Genes with the following criteria were designated as differentially-expressed for each major biopsy site (**Supplemental Figure S6C**): adjusted $p \leq 0.05$ & average read count over all 180 samples ≥ 50 and a $\log_2$ fold change < 0.1 (up-regulated in biopsy site). In downstream analyses, we marked these genes as “putative biopsy site-associated genes” (n = 7527) and discarded these as candidates. Masking these genes and performing an

identical t-SNE analysis on all 180 RNA-sequenced samples revealed reduced contribution of biopsy site (**Supplemental Figure S6E**).

Subsequently, we performed differential expression analysis using DESeq2 (v.1.24.0; Wald method) for CPCT-02 RNA-sequenced samples which were also included into the *CIRCUS* study and of which the date of biopsy and androgen measurement was less than 30 days apart, using total active androgen concentration as a continuous variable (n=15; **Supplemental Figure S7A**). Genes with the following criteria (and not present in the aforementioned "putative batch-effect genes"; **Supplemental Figure S7B and C**) were considered to be differentially expressed (n = 24): $|\log_2$ fold change$| \geq 0.5$ (with SE ≤ 1.25) & adjusted p ≤ 0.05 and an average read count ≥ 50 over all 15 samples.

Supplemental Table T1 – Baseline steroid concentrations of patients with or without exogenous glucocorticoids.

Undetectable concentrations were set to 0.5 times the lowest accurate calibration sample for statistical purposes and are italicized below. All concentrations are in nmol/L.

Abbreviations: IQR – interquartile range.

Steroids	Without glucocorticoids (n = 19)		With glucocorticoids (n = 15)		Median Difference (%)
	median (IQR)	Range	Median (IQR)	Range	
11-ketoandrostenedione	0.64 (0.43 - 1.07)	0.14 - 4.29	0.09 (0.04 - 0.22)	*0.03* - 0.96	86
11-ketotestosterone	0.87 (0.40 - 1.34)	0.12 - 2.39	0.12 (0.05 - 0.19)	*0.03* - 0.83	86
11β-hydroxyandrostenedione	4.96 (3.05 - 6.14)	0.86 - 18.77	0.41 (0.12 - 0.95)	*0.08* - 3.26	92
11β-hydroxytestosterone	0.23 (0.11 - 0.40)	*0.08* - 1.03	*0.08* (*0.08* - 0.09)	*0.08* - 0.26	66
Corticosterone	3.6 (2.5 – 6.0)	1.15 - 27.57	0.42 (0.43 - 1.90)	0.18 - 3.69	83
Cortisol	257.5 (199.3 - 326.9)	166.5 - 949.1	23.8 (18.2 - 65.8)	11.46 - 135.5	91
Cortisone	43.68 (34.26 - 58.44)	24.88 - 124.1	3.19 (0.34 - 11.56)	*0.07* - 20.71	93
Dihydrotestosterone	*0.05* (*0.05* - 0.07)	*0.05* - 0.27	*0.05* (*0.05* - *0.05*)	*0.05* - 0.07	0
Testosterone	0.23 (0.12 - 0.34)	0.05 - 0.76	0.06 (*0.02* - 0.12)	*0.02* - 0.15	74

Supplemental Table T2 – Overview of steroids and their mass transitions, retention times and analytical limits of quantification.

Abbreviations: LOQ – limits of quantification, m/z – mass per charge

Analyte	Abbreviation	Quantifier Ion (m/z)	Qualifier Ion (m/z)	Retention Time	Internal Standard	LOQ (nmol/L)
11-ketoandrostenedione	11KA4	301.0 > 121.0	301.0 > 265.2	1.11	11-ketotestosterone-d3	0.5
11-ketotestosterone	11KT	303.1 > 121.0	303.1 > 259.1	1.25	11-ketotestosterone-d3	0.2
11β-hydroxyandrostenedione	11OHA4	303.1 > 285.1	303.1 > 267.1	1.40	11β-hydroxyandrostenedione-d7	0.5
11β-hydroxytestosterone	11OHT	305.1 > 269.2	205.2 > 121.0	1.56	11β-hydroxyandrostenedione-d7	0.5
Corticosterone	-	347.1 > 329.1	347.1 > 121.0	1.65	Corticosterone-d8	0.2
Cortisol	-	363.1 > 121.0	363.1 > 90.9	1.18	Cortisol-d4	0.1
Cortisone	-	361.0 > 121.0	361.0 > 163.0	1.03	Cortisone-d7	0.1
Dihydrotestosterone	DHT	291.1 > 255.1	291.1 > 159.0	3.03	DHT-d3	0.2
Testosterone	T	289.1 > 96.9	289.1 > 109.0	2.38	Testosterone-d3	0.1

Supplemental Figure 1 – Association between circulating androgens and 11-oxygenated androgens in CRPC patients at baseline

Correlation was calculated using concentrations of all baseline samples (n=34).

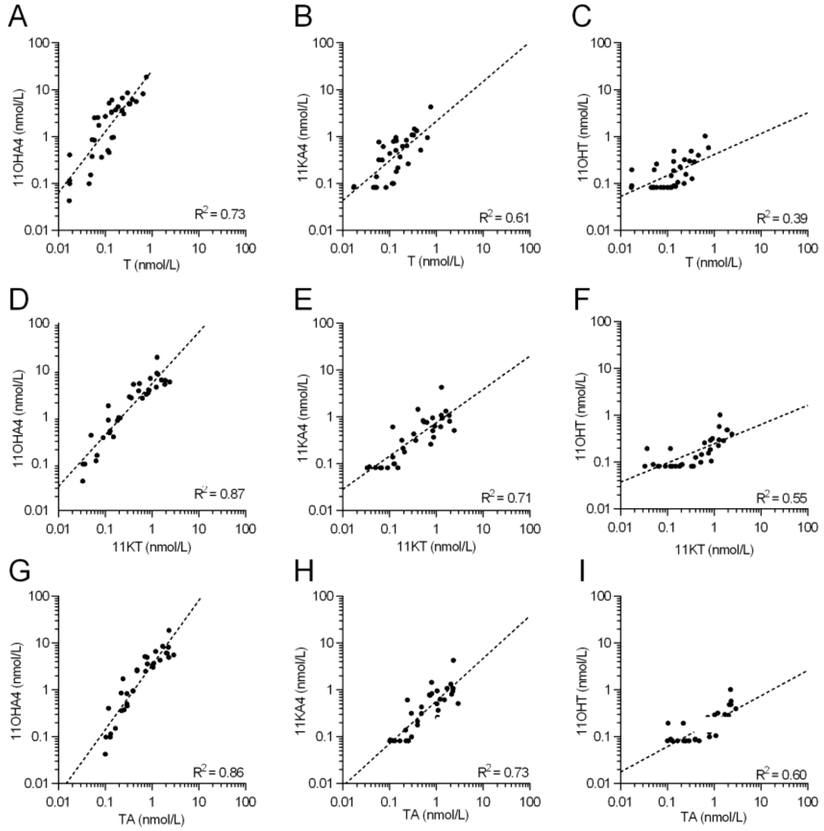

Abbreviations: 11KA4 – 11-ketoandrostenedione, 11KT – 11-ketotestosterone, 11OHA4 - 11β-hydroxyandrostenedione, 11OHT - 11β-hydroxytestosterone, T – testosterone, TA – total active androgens

Supplemental Figure S2 – **Baseline circulating steroid concentrations and effects of 12-week treatment.**

Steroid profiles were obtained for all castration-resistant prostate cancer patients before the start of first treatment (**A,** n=29). Steroid profiles at baseline and on treatment are shown for CRPC patients treated with AR antagonists (**B**, n=10), docetaxel with prednisone (**C**, n=10) and cabazitaxel with prednisone (**D**, n=14). Samples with undetectable concentrations were set to 0.5 times the lowest accurate calibration sample for statistical purposes. Differences between baseline (clear boxes) and on treatment (striped boxes) were assessed by Wilcoxon signed-rank test. Boxplot depicts the upper and lower quartiles, with the median shown as a solid line; whiskers indicate the range. * $p<0.05$, ** $p<0.01$.

Boxes are colored to reflect steroid synthesis pathways: mineralocorticoid (red), glucocorticoid (yellow), androgen (blue) and 11-oxygenated androgen (green). Lighter colors are used to indicate precursor steroids.

Abbreviations: CRPC – castration-resistant prostate cancer,

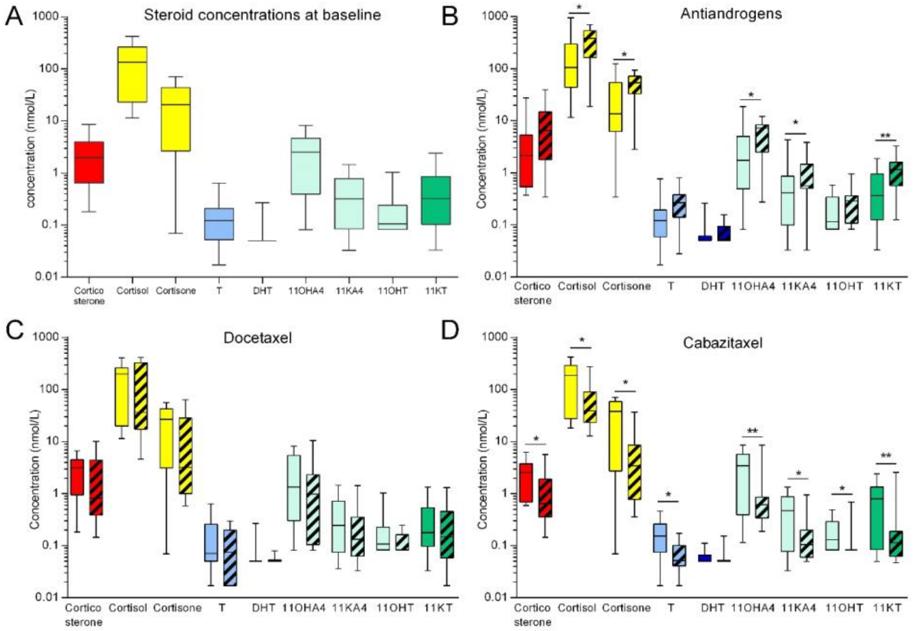

A
Steroid concentrations at baseline
B
Antiandrogens
C
Docetaxel
D
Cabazitaxel
concentration (nmol/L)
Concentration (nmol/L)
1000
100
10
1
0.1
0.01
Cortico sterone
Cortisol
Cortisone
T
DHT
11OHA4
11KA4
11OHT
11KT

Supplemental Figure S3 – Quantification of prednisone, prednisolone and dexamethasone identifies glucocorticoid suppressed patient samples.

Quantification of prednisone (**A)**, prednisolone (**B)** and dexamethasone (**C**) was performed using LC-MS/MS to identify the use of exogenous glucocorticoids in patient samples for classification. Values that were obtained during this measurement should be considered in the context of this study only, as appropriate internal standards had not been included in the initial extraction. No suppression of adrenal steroids was observed in patients with exogenous glucocorticoid values below the red lines. Classification of the exogenous glucocorticoids successfully identified samples with suppressed cortisol values at baseline (**D**).

Abbreviations: GC - glucocorticoid

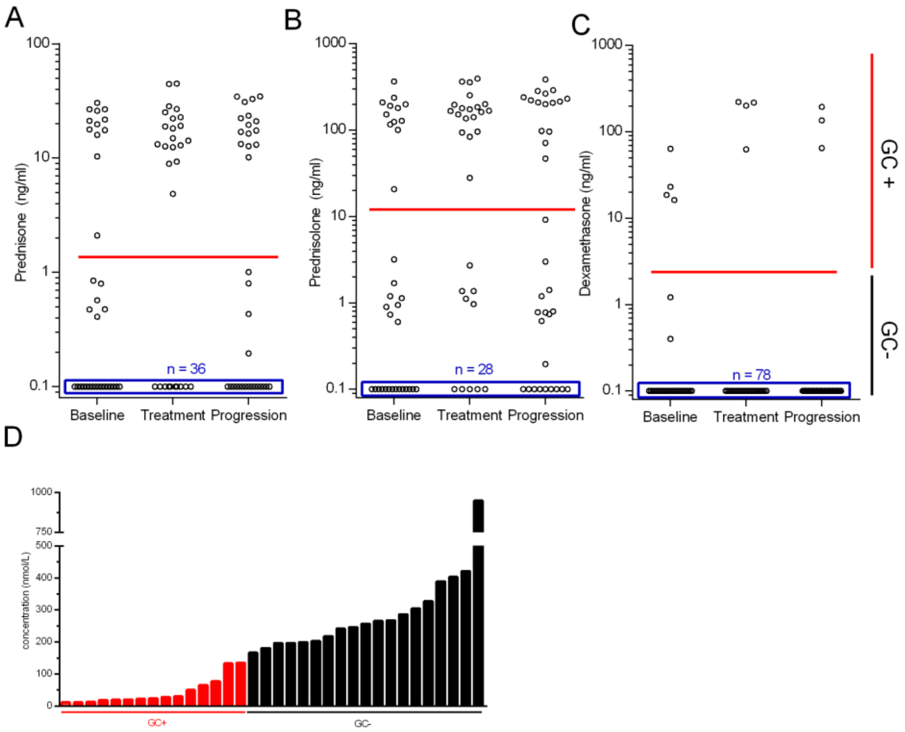
A
B
C
D
Prednisone (ng/ml)
Prednisolone (ng/ml)
Dexamethasone (ng/ml)
n = 36
n = 28
n = 78
Baseline
Treatment
Progression
GC +
GC-
concentration (nmol/L)
GC+
GC-

Supplemental Figure S4 – **Circulating androgens are higher in patients withdrawn before progression.** The effects of glucocorticoid withdrawal were studied in patients who were exogenous glucocorticoid treated on treatment (n=20) (**A**). Differences between steroid concentrations upon progression were assessed in patients in whom glucocorticoid treatment was continued (gray boxes, n=14) or discontinued (blue boxes, n=6) by Wilcoxon signed-rank test. Boxplot depicts the upper and lower quartiles, with the median shown as a solid line; whiskers indicate the range. * $p<0.05$, ** $p<0.01$.

The individual data points are shown for testosterone (T, **B**) and 11-ketotestosterone (11KT, **C**) for patients that received exogenous glucocorticoids on treatment and continued (gray lines, n=14) or discontinued glucocorticoid treatment (blue lines, n=6). Samples with undetectable concentrations were set to 0.5 times the lowest accurate calibration sample for statistical purposes. Effects of treatment were assessed by Wilcoxon ranked-sum test, while group differences were assessed by Mann-Whitney test. Lines connect individual patients and group medians (squares) are shown beside the individual data points. ** $p<0.01$.

Abbreviations: 11KA4 – 11-ketoandrostenedione, 11KT – 11-ketotestosterone, 11OHA4 - 11β-hydroxyandrostenedione, 11OHT - 11β-hydroxytestosterone, DHT – dihydrotestosterone, OT – on treatment, PD – progressive disease, T – testosterone

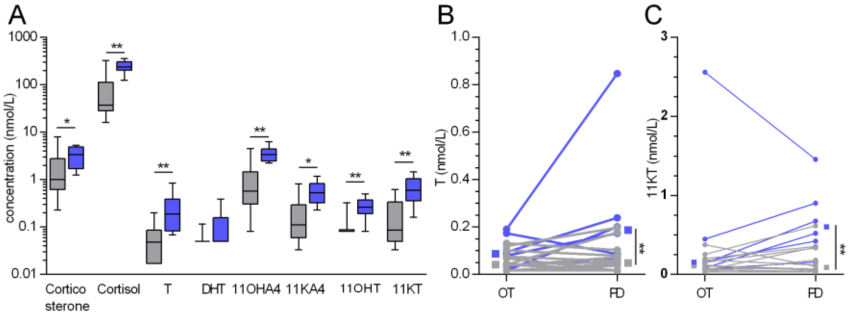
A
1000
100
10
1
0.1
0.01
concentration (nmol/L)
*
**
**
**
*
**
**
Cortico sterone
Cortisol
T
DHT
11OHA4
11KA4
11OHT
11KT
B
1.0
0.8
0.6
0.4
0.2
0.0
T (nmol/L)
**
OT
FD
C
3
2
1
0
11KT (nmol/L)
**
OT
FD

Supplemental Figure S5 - **11-oxygenated androgen precursors are not associated with progression-free survival.**

Progression-free survival (PFS) of patients was stratified according to concentrations above or below the median.

Abbreviations: 11KA4 – 11-ketoandrostenedione, 11OHA4 - 11β-hydroxyandrostenedione, 11OHT - 11β-hydroxytestosterone, PFS – progression-free survival.

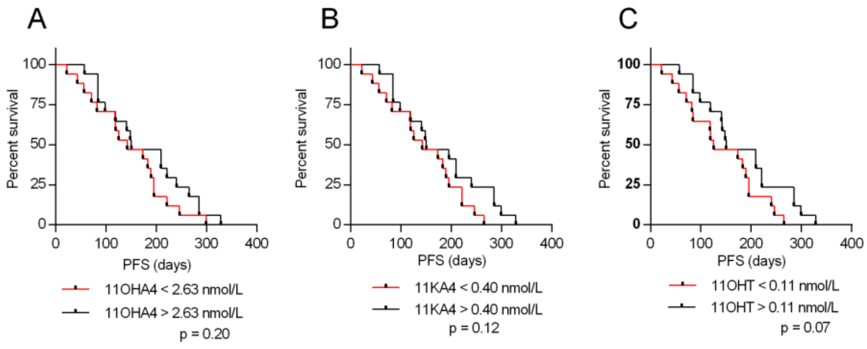

Supplemental Figure S6 - Overview of the CPCT-02 (m)RNA-sequenced metastatic prostate cancers (*n*= 180) including selection of biopsy site-specific expression.

(**A**) Sequencing and alignment metrics (flagstat) highlighting samples (*n* = 180) performed on the Illumina NextSeq (orange) and Illumina NovaSeq (blue) platform. Y-axis depicts number of reads per sample and X-axis depicts various alignment metrics. Boxplot depicts the upper and lower quartiles, with the median shown as a solid line; whiskers indicate 1.5 times the interquartile range. Individual data points are shown. Median per alignment metric (per million) is shown on the left-side of each boxplot.

(**B**) Number of RNA-sequenced samples per biopsy site. Biopsy sites with fewer than 3 samples were categorized into the 'Other' category. Y-axis depicts number of samples and X-axis depicts the major biopsy sites (bone, lymph node and liver). Number of samples is shown above each bar per biopsy site.

(**C**) Venn-diagram of differentially expressed protein-coding genes (adjusted $p \leq 0.05$ & average read count over all 180 samples ≥ 50 and a log2 fold change < 0.1) per biopsy site.

(**D**) t-SNE analysis of all 180 samples over the read counts of all protein-coding genes (*n* = 20084). Samples are colored per biopsy site and shaped per sequencing platform (circle for Illumina NextSeq and triangle for Illumina NovaSeq).

(**E**) t-SNE analysis of all 180 samples over the read counts of all protein-coding genes (*n* = 20084), except those marked as "putative biopsy site associated genes" (*n* = 7527). Samples are colored per biopsy site and shaped per sequencing platform (circle for Illumina NextSeq and triangle for Illumina NovaSeq).

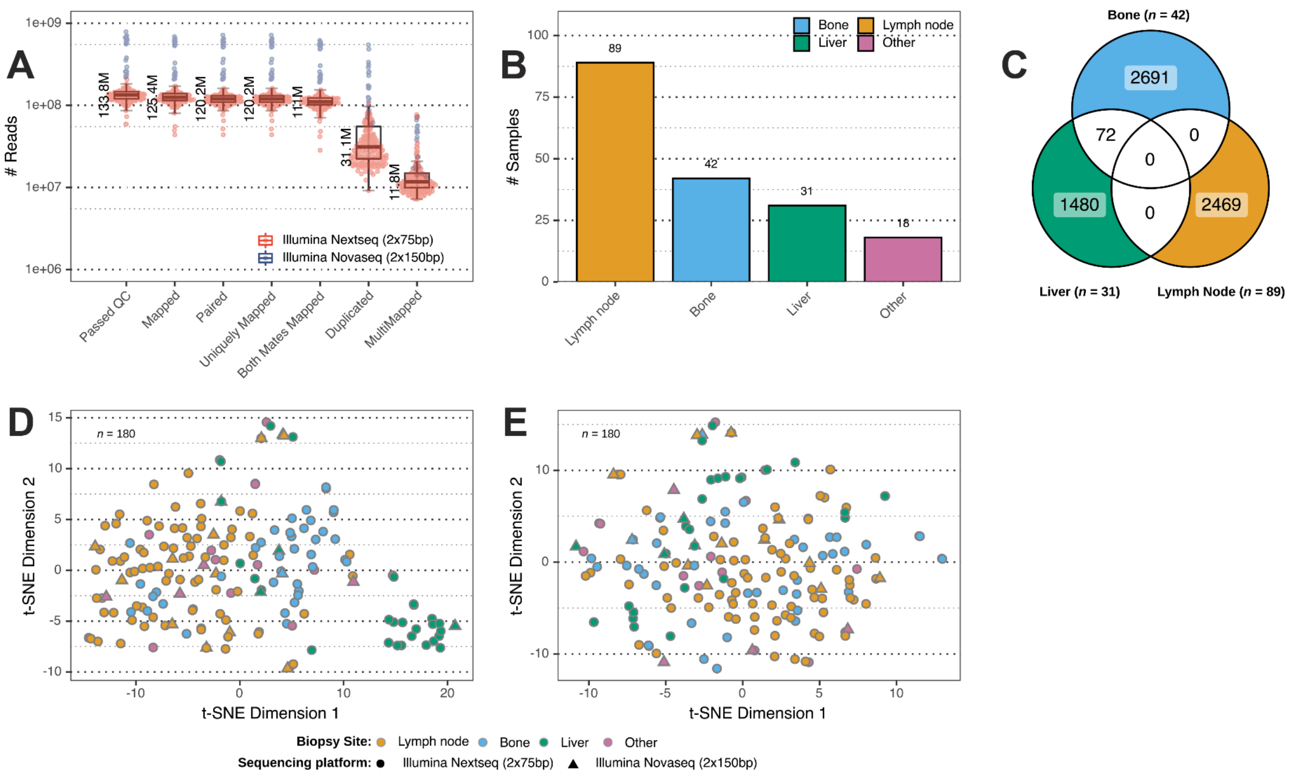
A
B
C
D
E
Reads
133.8M
125.4M
120.2M
120.2M
111M
31.1M
11.8M
Illumina Nextseq (2x75bp)
Illumina Novaseq (2x150bp)
Passed QC
Mapped
Paired
Uniquely Mapped
Both Mates Mapped
Duplicated
MultiMapped
Samples
Bone
Lymph node
Liver
Other
89
42
31
18
Bone (n = 42)
Liver (n = 31)
Lymph Node (n = 89)
2691
72
0
0
0
1480
2469
n = 180
t-SNE Dimension 1
t-SNE Dimension 2
Biopsy Site: Lymph node Bone Liver Other
Sequencing platform: Illumina Nextseq (2x75bp) Illumina Novaseq (2x150bp)

Supplemental Figure S7 – Overview of the CPCT-02 (m)RNA-sequenced metastatic prostate cancers (n=15) present in the *CIRCUS* study.

- **(A)** Overview of the inclusion criteria of *CIRCUS* samples present within the CPCT-02 RNA-sequenced metastatic prostate cancer cohort (n = 180) and categorization of these samples (n=15) into androgen status high and low samples.
- **(B)** t-SNE analysis of the 15 included samples over the read counts of all protein-coding genes (n=20084). Samples are colored per biopsy site and shaped per androgen status (circle for high androgen status and triangle for low androgen status).
- **(C)** t-SNE analysis of all 15 samples over the read counts of all protein-coding genes (n=20084), except those marked as "putative biopsy site-associated genes" for lymph node or bone (n=5232). Genes associated with the liver biopsy site were not excluded (n=1480) as none of the biopsies originated from liver metastasis. Samples are colored per biopsy site and shaped per androgen status (circle for high androgen status and triangle for low androgen status).

Abbreviations, Enza – enzalutamide, GC – glucocorticoid, RNA-seq - RNA sequencing, TA – total active androgens

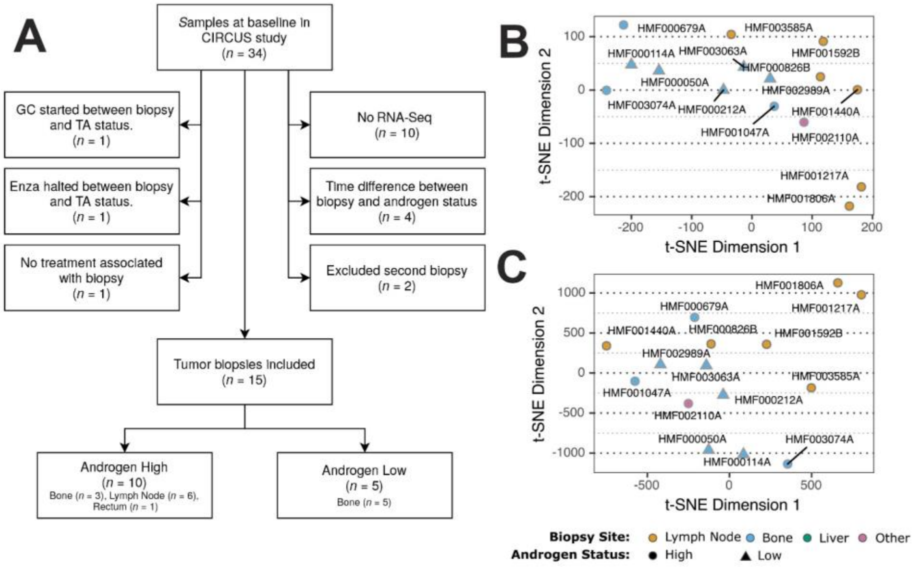
A
Samples at baseline in CIRCUS study (n = 34)
GC started between biopsy and TA status. (n = 1)
Enza halted between biopsy and TA status. (n = 1)
No treatment associated with biopsy (n = 1)
No RNA-Seq (n = 10)
Time difference between biopsy and androgen status (n = 4)
Excluded second biopsy (n = 2)
Tumor biopsies included (n = 15)
Androgen High (n = 10)
Bone (n = 3), Lymph Node (n = 6), Rectum (n = 1)
Androgen Low (n = 5)
Bone (n = 5)
B
C
t-SNE Dimension 1
t-SNE Dimension 2
Biopsy Site: Lymph Node Bone Liver Other
Androgen Status: High Low

Supplemental Figure S8 - Intratumoral expression of genes involved in steroid metabolism and steroid hormone receptors in metastatic castration-resistant prostate cancer.

Normalized (VST-transformed) RNA-seq read counts of steroid hormone receptors and enzymes involved in the conversion and metabolism of steroid hormones. Boxplot depicts the upper and lower quartiles, with the median shown as a solid line; whiskers indicate 1.5 times the interquartile range. Individual data points are shown.

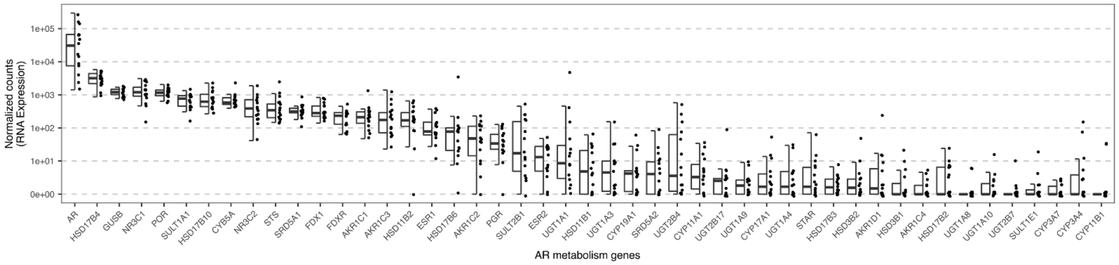

Supplemental Figure S9 – Chromatographic separation of endogenous steroids.

Representative chromatographic separation of 9 steroids: (1) cortisone, (2) 11-ketoandrostenedione, (3) cortisol, (4) 11-ketotestosterone, (5) 11β-hydroxyandrostenedione, (6) 11β-hydroxytestosterone, (7) corticosterone, (8) testosterone, (9) dihydrotestosterone. The top chromatogram shows separation with normalized intensity, whereas bottom graph shows actual response.

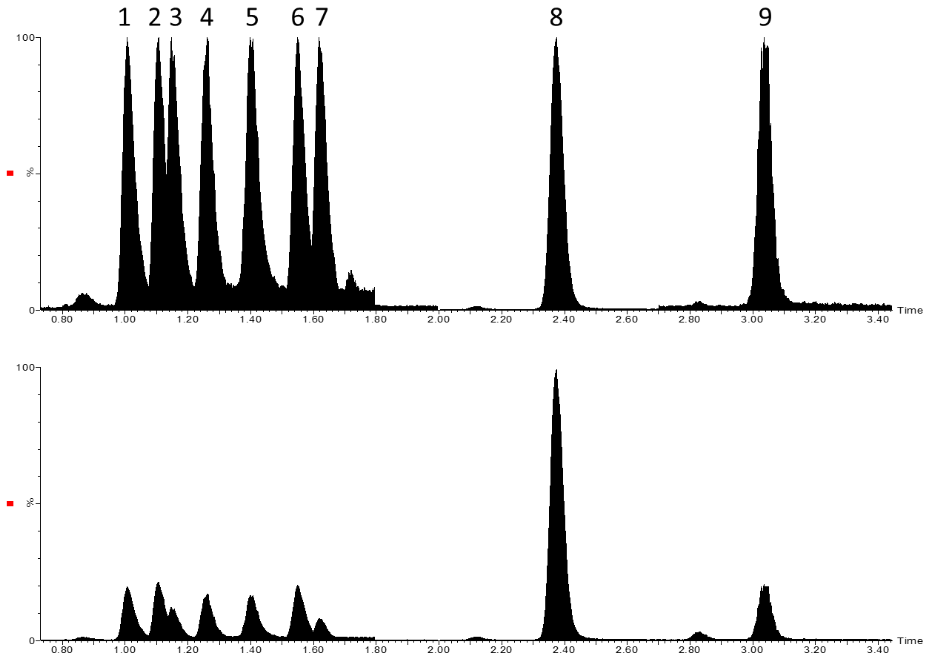

References (Supplementary)

1. van der Pas R, et al. Fluconazole inhibits human adrenocortical steroidogenesis in vitro. *J Endocrinol.* 2012;215(3):403-12.
2. O'Reilly MW, et al. 11-Oxygenated C19 Steroids Are the Predominant Androgens in Polycystic Ovary Syndrome. *J Clin Endocrinol Metab.* 2017;102(3):840-8.
3. O'Reilly MW, et al. Hyperandrogenemia predicts metabolic phenotype in polycystic ovary syndrome: the utility of serum androstenedione. *J Clin Endocrinol Metab.* 2014;99(3):1027-36.
4. Quanson JL, et al. High-throughput analysis of 19 endogenous androgenic steroids by ultra-performance convergence chromatography tandem mass spectrometry. *J Chromatogr B Analyt Technol Biomed Life Sci.* 2016;1031:131-8.
5. Snaterse G, et al. Validation of circulating steroid hormone measurements across different matrices by liquid chromatography-tandem mass spectrometry. *Steroids.* 2021.
6. Pretorius E, et al. 11-Ketotestosterone and 11-Ketodihydrotestosterone in Castration Resistant Prostate Cancer: Potent Androgens Which Can No Longer Be Ignored. *PLoS One.* 2016;11(7):e0159867.
7. Hassan-Smith ZK, et al. Gender-Specific Differences in Skeletal Muscle 11beta-HSD1 Expression Across Healthy Aging. *J Clin Endocrinol Metab.* 2015;100(7):2673-81.
8. Richards J, et al. Interactions of abiraterone, eplerenone, and prednisolone with wild-type and mutant androgen receptor: a rationale for increasing abiraterone exposure or combining with MDV3100. *Cancer Res.* 2012;72(9):2176-82.
9. Chen S, et al. fastp: an ultra-fast all-in-one FASTQ preprocessor. *Bioinformatics.* 2018;34(17):i884-i90.
10. Dobin A, et al. STAR: ultrafast universal RNA-seq aligner. *Bioinformatics.* 2013;29(1):15-21.
11. Harrow J, et al. GENCODE: the reference human genome annotation for The ENCODE Project. *Genome Res.* 2012;22(9):1760-74.
12. Tarasov A, et al. Sambamba: fast processing of NGS alignment formats. *Bioinformatics.* 2015;31(12):2032-4.
13. Love MI, et al. Moderated estimation of fold change and dispersion for RNA-seq data with DESeq2. *Genome Biol.* 2014;15(12):550.
14. Laurens Van Der M. Accelerating t-SNE using tree-based algorithms. *J Mach Learn Res.* 2014;15(1):3221–45.
15. Stephens M. False discovery rates: a new deal. *Biostatistics.* 2016;18(2):275-94.
